# Three new *Scheffersomyces* species associated with insects and rotting wood in China

**DOI:** 10.3897/mycokeys.71.56168

**Published:** 2020-08-05

**Authors:** Ran-Ran Jia, Shi-Long Lv, Chun-Yue Chai, Feng-Li Hui

**Affiliations:** 1 School of Life Science and Technology, Nanyang Normal University, Nanyang 473061, China

**Keywords:** *
Debaryomycetaceae
*, *
Saccharomycetales
*, taxonomy, d-xylose-fermenting yeast

## Abstract

Three species of *Scheffersomyces* were identified during a diversity study of yeasts. All three are associated with insects and rotting wood in China. Phylogenetic analyses of a genomic dataset combining ITS and nrLSU revealed that these new collections are distinct from known species, thus three new species are introduced i.e. *S. jinghongensis*, *S. paraergatensis*, and *S. anoplophorae*. In our phylogenetic analyses, *Scheffersomyces jinghongensis* possesses a strong independent lineage and is closely related to *S. titanus*. *S. paraergatensis* is closely related to *S. ergatensis*, while *S. anoplophorae* is related to *S. stambukii.* Several differences in physiological traits and molecular data indicate that *S. jinghongensis*, *S. paraergatensis*, and *S. anoplophorae* are three newly identified species.

## Introduction

Kurtzman and Suzuki (2010) introduced the genus *Scheffersomyces* Kurtzman. ex M. Suzuki. (2010) to include d-xylose-fermenting species in the *Pichia stipitis* clade, viz. *P. segobiensis*, *P. spartinae*, and *P. stipitis*. The genus *Scheffersomyces* was later expanded by including seven related *Candida* species as new combinations and by three novel species, *S. illinoinensis*, *S. quercinus*, and *S. virginianus*, which were discovered in rotting wood (Urbina and Blackwell 2012). More recently, several new species and combinations from the genus *Scheffersomyces*, (*S. cryptocerci*, *S. parashehatae*, *S. titanus*, and *S. xylosifermentans*) have been identified in wood-ingesting insects (Suh et al. 2013; Urbina et al. 2013; Liu et al. 2016b), and in rotting wood (*S. amazonensis*, *S. ergatensis*, *S. henanensis*, and *S. stambukii*) (Ren et al. 2013; Urbina and Blackwell 2013; Lopes et al. 2018). This genus comprises 21 species, though the status of *S. gosingicus* and *S. lignicola* is currently unclear due to nomenclatural issues regarding the Melbourne Code (Index Fungorum, accessed 17.07.2020). Of the 19 valid species, five of them are known to form ascospores (Kurtzman 2011; Ren et al. 2013, Liu et al. 2016b) and 14 of them display asexual morphs (Urbina and Blackwell 2012; Suh et al. 2013; Urbina and Blackwell 2013; Urbina et al. 2013; Lopes et al. 2018).

Species in the genus *Scheffersomyces* are characterized by pseudohyphae formation, an inability to utilize nitrates, and the possession of the co-enzyme Q-9 (Kurtzman and Suzuki 2010; Kurtzman 2011). Asexual reproduction occurs via multilateral budding on a narrow base; sexual reproduction occurs via the formation of 1–2 hat-shaped ascospores released soon after formation (Kurtzman 2011). Several *Scheffersomyces* species, such as *S. henanensis*, *S. shehatae*, and *S. stipitis*, strongly ferment d-xylose, which is important for producing bioethanol from the residue of plant waste (du Preez and van der Walt 1983; Agbogbo and Wenger 2006; Ren et al. 2013; Suh et al. 2013; Lopes et al. 2018). Despite the existence of these microbes, obtaining high ethanol yields from pentose sugars on a large scale remains a challenge (Chandel et al. 2011) because scientists have yet to identify microbes that convert pentose sugars into ethanol at high yields, while withstanding fermentation inhibitors (Agbogbo and Wenger 2006; Long et al. 2012). Therefore, there is a need to identify new yeasts capable of efficient xylose fermentation in order to produce bioethanol.

Several d-xylose-fermenting yeasts were collected from different regions in China during a study on fungal diversity in insects and rotting wood. Two *Scheffersomyces* species, *S. henanensis* and *S. titanus*, have already been described in published studies (Ren et al. 2013; Liu et al. 2016b). In this study, the other three new species are described and characterized based on their morphology and phylogenetics, increasing the species diversity of *Scheffersomyces* in China.

## Materials and methods

### Sample collection, morphological studies, and isolation

Samples of insects and rotting wood were collected from Henan Province and Yunnan Province from 2015–2017. Strains of yeast were isolated from the insect guts according to the methods described by Liu et al. (2016a, b). Prior to dissection, the insects were placed in Petri dishes for 1–3 days without food, which eliminates some of the contaminating organisms found in the gut. Surface disinfection was performed by submersion in 95% ethanol for 1–2 min, followed by rinsing in a 0.7% saline solution. The insect gut was removed aseptically under a dissecting microscope. The gut segments were streaked on acidified yeast extract–malt extract (YM) agar plates (0.3% yeast extract, 0.3% malt extract, 0.5% peptone, 1% glucose, 2% plain agar; pH adjusted to 3.5 with HCl) and incubated at 25 °C for 3–4 days. Strains were isolated from the samples of rotting wood via the enrichment technique with YM broth, supplemented by 0.025% sodium propionate and 0.01% chloramphenicol (Ren et al. 2013). All yeast isolates were purified by repeated streak-inoculation on YM agar plates and preserved in 15% glycerol at -80 °C.

The morphological, physiological, and biochemical properties were determined according to those used by Kurtzman et al. (2011). The beginning of the sexual stage was determined by incubating single or mixed cultures of each of the two strains on cornmeal (CM) agar, YM agar, or 5% malt extract (ME) agar at 25 °C for 6 weeks (Ren et al. 2013, Liu et al. 2016b). The assimilation of carbon and nitrogen compounds and related growth requirements were tested at 25 °C. The effects of temperature from 25–40 °C were examined in a liquid culture and on agar plates.

### DNA extraction, PCR amplification, and sequencing

Genomic DNA was extracted from the yeast using an Ezup Column Yeast Genomic DNA Purification Kit, according to the manufacturer’s instructions (Sangon Biotech, Shanghai, China). The nuc rDNA ITS1-5.8S-ITS2 (ITS) region was amplified using primer pairs ITS1/ITS4 (White et al. 1990). The D1/D2 domain of nrLSU rDNA (nrLSU) was amplified using the primer pair NL1/NL4 (Kurtzman and Robnett 1998). The PCR protocols used for the ITS and nrLSU were those outlined by Liu et al. (2016a). PCR products were directly purified and sequenced by Sangon Biotech Inc. (Shanghai, China). We confirmed the identity and accuracy of the resulting sequences by assembling them using BioEdit and comparing them to sequences in GenBank (Hall 1999). The sequences were then submitted to GenBank (https://www. ncbi.nlm.nih.gov/genbank/; Table 1).

**Table 1.** Sequences used in molecular phylogenetic analysis. Entries in bold are newly generated for this study.

Species	Strain	ITS	D1/D2
*Candida broadrunensis*	CBS 11838^T^	HQ263349	NG_064320
*Candida thasaenensis*	CBS 12529^T^	NR_111028	NG_055174
***Scheffersomyces anoplophorae***	**NYNU 15730^T^**	**KU128714**	**KU128724**
***Scheffersomyces anoplophorae***	**NYNU 15733**	**MT133542**	**MT133540**
*Scheffersomyces coipomoensis*	NRRL Y-17651^T^	HQ652070	HQ651966
*Scheffersomyces cryptocerci*	CBS 12658^T^	NR_120091	NG_055704
*Scheffersomyces ergatensis*	NRRL Y-17652^T^	EU343826	U45746
*Scheffersomyces gosingicus*	CBS 11433 ^T^	HQ999978	HQ999955
*Scheffersomyces henanensis*	CBS 12475 ^T^	HQ127627	HQ127626
*Scheffersomyces illinoinensis*	NRRL Y-48827^T^	JN943261	JN703959
*Scheffersomyces insectosa*	NRRL Y-12854^T^	NR_111587	NG_055695
***Scheffersomyces jinghongensis***	**NYNU 17926^T^**	**MG255722**	**MG255714**
***Scheffersomyces jinghongensis***	**NYNU 17977**	**MT133547**	**MT133543**
*Scheffersomyces lignicola*	CBS 10610 ^T^	HQ652074	AY845350
*Scheffersomyces lignosus*	NRRL Y-12856^T^	R_120020	NG_055694
***Scheffersomyces paraergatensis***	**NYNU 16782^T^**	**KY213803**	**KY213826**
***Scheffersomyces paraergatensis***	**NYNU 16969**	**MT133541**	**MT133546**
*Scheffersomyces parashehatae*.	CBS 12535^T^	NR_138230	NG_055697
*Scheffersomyces queiroziae*	NRRL Y-48722^T^	HM566445	HM566445
*Scheffersomyces quercinus*	NRRL Y-48825^T^	JN943260	JN703957
*Scheffersomyces segobiensis*	NRRL Y-11571^T^	NR_111217	NG_055696
*Scheffersomyces shehatae*	NRRL Y-12858^T^	JN943264	JQ025409
*Scheffersomyces spartinae*	NRRL Y-7322^T^	HQ876044	U45764
*Scheffersomyces stambukii*	CBS 14217^T^	KT033721	KT033720
*Scheffersomyces stipitis*	NRRL Y-7124^T^	JN943257	U45741
*Scheffersomyces titanus*	CBS 13926^T^	KP054263	KP054262
*Scheffersomyces virginianus*	NRRL Y-48822^T^	NR_120018	NG_055702
*Scheffersomyces xylosifermentans*	CBS 12540^T^	KY105362	KY109586
*Saccharomyces cerevisiae*	CBS 1171^T^	NR_111007	NG_042623

Abbreviations: **CBS**: CBS-KNAW Collections, Westerdijk Fungal Biodiversity Institute, Utrecht, The Netherlands; **NRRL**: the Agricultural Research Service Culture Collection, Peoria, IL, USA; **NYNU**: Microbiology Lab, Nanyang Normal University, Henan, China; **T**: type strain.

### Phylogenetic analysis

The sequences obtained from this study and the reference sequences obtained from GenBank (Table 1) were aligned using MAFFT v. 6 (Katoh and Toh 2010) and manually edited using MEGA v. 7 (Kumar et al. 2016). The best-fit nucleotide substitution models for each gene were selected using jModelTest v2.1.7 (Darriba et al. 2012) according to the Akaike information criterion. Phylogenetic analyses of combined gene regions (ITS and nrLSU) were performed using MEGA v7 for maximum parsimony (MP) analysis (Kumar et al. 2016) and PhyML v3.0 for Maximum Likelihood (ML) analysis (Guindon et al. 2010). *Saccharomyces cerevisiae* CBS 1171^T^ was chosen as the outgroup after consulting Liu et al. (2016b) and Suh et al. (2013).

MP analysis was performed using a heuristic search option with tree-bisection reconnection (TBR) branch swapping (Nei and Kumar 2000) and 1,000 random sequence additions. ML analysis was performed using GTR+I+G models for each partition (Nei and Kumar 2000) and a proportion of invariant sites with 1000 rapid bootstrap replicates. The phylogenies from MP and ML analyses were displayed using Mega 7 and FigTree v1.4.3 (Rambaut 2016), respectively. Bootstrap support values ≥ 50% are shown at the nodes.

## Results

**Figure 1.**
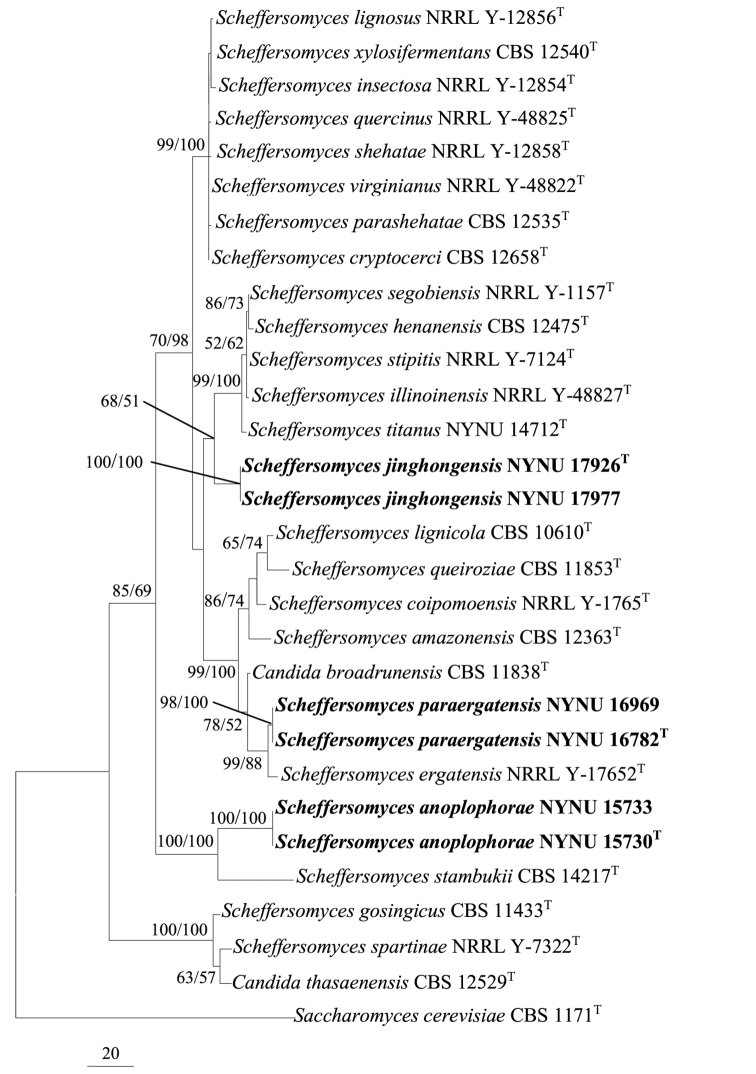
Maximum parsimony phylogenetic tree generated from analysis of combined ITS and nrLSU sequences dataset for 24 taxa of *Scheffersomyces* and related *Candida* species. *Saccharomyces cerevisiae* CBS 1171^T^ is the out-group taxon. Bootstrap values ≥ 50% for MP/ML analyses are presented at the branches. Scale bar = 20 nucleotide substitutions. The species from this study are indicated in bold letters.

The alignment was based on the combined sequence dataset (ITS and nrLSU) and included 26 in-group taxa and one out-group taxon (*Saccharomyces cerevisiae* CBS 1171^T^), comprised of 1085 characters in the aligned matrix. Of these, 541 characters were constant, 356 variable characters were parsimony-uninformative, and 188 characters were parsimony-informative. The MP analysis resulted in three equally parsimonious trees; the first tree (TL = 589, CI = 0.640, RI = 0.833, RC = 0.533) is shown in Fig. 1. The three MP trees were identical, except for the species order within different clades. ML analyses revealed that tree topologies of the best tree were identical to those of the MP tree (not shown). The sequences of each three new species formed a well-supported monophyletic group (MP = 98–100%, ML = 100%). *S. paraergatensis* and *S. anoplophorae* were related to *S. ergatensis* and *S. stambukii*, respectively, while *S. jinghongensis* possessed a strongly independent lineage that is distinct from other species (Fig. 1).

### Taxonomy

#### Scheffersomyces jinghongensis

Taxon classificationFungiSaccharomycetalesSaccharomycetaceae

C.Y. Chai & F.L. Hui sp. nov.

EEE03D3A-E86D-5572-83EA-EE537A01F970

MycoBank No: 835004

Figure 2

##### Etymology.

The species name *jinghongensis* (N.L. fem. adj.) refers to the geographical origin of the type strain of this species.

##### Holotype.

NYNU 17926^T^.

##### Isolation data.

China, Yunnan Province, Jinghong, in rotting wood, under a tropical rainforest, July 2017, K.F. Liu & Z.W. Xi (ex-holotype: CICC 33270; CBS 15230).

##### Description.

**Figure 2.**
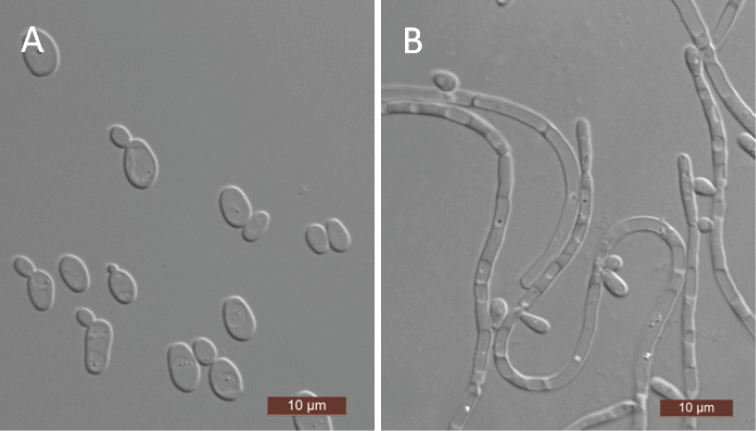
Morphology of *S. jinghongensis*. **A** budding cells **B** pseudohyphae. Scale bars: 10 μm.

The cells are ovoid to elongate (3–4 ×3–7.5 μm) and occur singly or in pairs after being placed in YM broth for 3 days at 25 °C (Fig. 2A). Budding is multilateral. After 3 days of growth on YM agar at 25 °C, the colonies are white to cream-colored, buttery, and smooth, with entire margins. After 7 days at 25 °C on a Dalmau plate culture with CM agar, pseudohyphae were observed but true hyphae were not (Fig. 2B). Asci or signs of conjugation were not observed on sporulation media. Glucose, galactose, trehalose, cellobiose, and d-xylose (weak) are fermented, but maltose, sucrose, melibiose, lactose, melezitose, raffinose, and inulin are not. Glucose, galactose, l-sorbose, d-glucosamine, d-ribose, d-xylose, sucrose, maltose, trehalose, cellobiose, arbutin, melezitose, inulin, glycerol, erythritol, ribitol, xylitol, d-glucitol, d-mannitol, d-glucono-1, 5-lactone, 2-keto-d-gluconate, d-glucuronate, succinate, and ethanol are assimilated. No growth was observed in l-arabinose, d-arabinose, l-rhamnose, methyl α-d-glucoside, salicin, melibiose, lactose, raffinose, l-arabinitol, galactitol, *myo*-inositol, 5-keto-d-gluconate, d-gluconate, dl-lactate, citrate, or methanol. For the assimilation of nitrogen compounds, growth on l-lysine, glucosamine, or d-tryptophan is positive, while growth on nitrate, nitrite, ethylamine, cadaverine, creatine, creatinine, or imidazole is negative. Growth is observed at 37 °C but not at 40 °C. Growth in the presence of 0.1% cycloheximide is positive, but growth in the presence of 10% NaCl with 5% glucose and 1% acetic acid is negative. Starch-like compounds are not produced. Urease activity and diazonium blue B reactions are also negative.

##### Additional isolate examined.

China, Yunnan Province, Jinghong, in rotting wood, under a tropical rainforest, July 2017, K.F. Liu & Z.W. Xi, NYNU 17977.

##### GenBank accession numbers.

holotype NYNU 17926^T^ (ITS:MG255722; nrLSU D1/D2: MG255714) ; additional isolate NYNU 17977 (ITS: MT133547; nrLSU D1/D2: MT133543).

##### Notes.

Two strains representing *S. jinghongensis* were grouped in an independent lineage and are related to *S. titanus* and other *Scheffersomyces* species. The nucleotide differences between the new species and the close relative *S. titanus* (Liu et al. 2016b) are 1.6% substitutions in the D1/D2 domain and 4.9% substitutions in the ITS region, respectively. Physiologically, *S. jinghongensis* can be differentiated from *S. titanus* based on growth in l-arabinose, l-rhamnose, methyl α-d-glucoside, salicin, lactose, *myo*-inositol, and 5-keto-d-gluconate, all of which were positive for *S. titanus* and negative for the new species. Additionally, *S. jinghongensis* ferments cellobiose and grows at 37 °C, but not for *S. titanus*.

#### Scheffersomyces paraergatensis

Taxon classificationFungiSaccharomycetalesSaccharomycetaceae

C.Y. Chai & F.L. Hui sp. nov.

9DA59AA2-17B5-5802-B586-3066E6585D57

MycoBank No: 835005

Figure 3

##### Etymology.

The species name *paraergatensis* (Gr. prep.) refers to its phylogenetic similarity to *S. ergatensis*.

##### Holotype.

NYNU 16782^T^.

##### Isolation data.

China, Henan Province, Nanyang, in rotting wood, under a mixed forest, July 2016, K.F. Liu & Z.W. Xi (ex-holotype: CICC 33165; CBS 14694).

##### Description.

**Figure 3.**
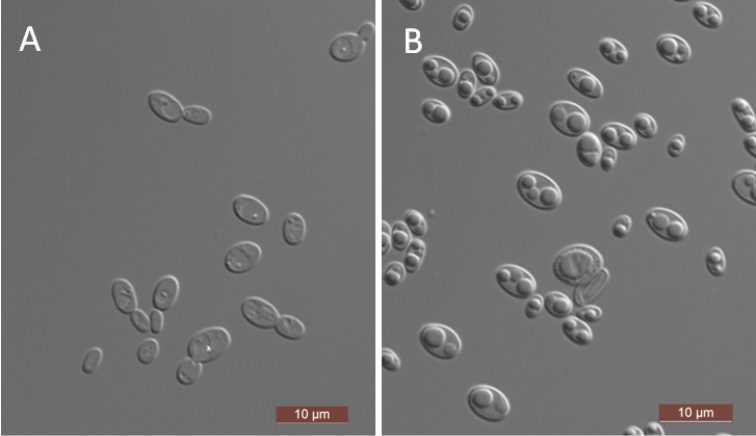
Morphology of *S. paraergatensis*. **A** budding cells **B** ascus and ascospores. Scale bars: 10 μm.

The cells are ovoid to elongate (2.5–5×3.5–6 μm) and occur singly or in pairs after grown in a YM broth for 3 days at 25 °C (Fig. 3A). Budding is multilateral. After 3 days of growth on YM agar at 25 °C, the colonies are white to cream-colored, buttery, and smooth with entire margins. After 7 days at 25 °C, on a Dalmau plate culture with CM agar, pseudohyphae were observed but true hyphae were not. Conjugated asci formed after 6 days at 25 °C on CM agar and 5% ME agar, with each ascus containing one or two hat-shaped ascospores (Fig. 3B). Glucose, galactose, and d-xylose are weakly fermented, but maltose, sucrose, trehalose, melibiose, lactose, cellobiose, melezitose, raffinose, and inulin are not. Glucose, galactose, d-ribose, d-xylose, l-arabinose, d-arabinose, sucrose, maltose, trehalose, methyl α-d-glucoside, cellobiose, salicin, arbutin, lactose, raffinose, inulin, glycerol, ribitol, xylitol, d-glucitol, d-mannitol, d-glucono-1, 5-lactone, d-gluconate, succinate, citrate, and ethanol are assimilated. No growth was observed in l-sorbose, d-glucosamine, l-rhamnose, melibiose, melezitose, erythritol, galactitol, *myo*-inositol, 2-keto-d-gluconate, 5-keto-d-gluconate, d-glucuronate, dl-lactate, or methanol. For the assimilation of nitrogen compounds, growth on l-lysine, glucosamine, and d-tryptophan is positive, while growth on nitrate, nitrite, ethylamine, cadaverine, creatine, creatinine, and imidazole is negative. Growth was observed at 30 °C, but not at 35 °C. Growth in the presence of 0.1% cycloheximide is positive, but growth in the presence of 10% NaCl with 5% glucose and 1% acetic acid is negative. Starch-like compounds are not produced. Urease activity and diazonium blue B reactions are also negative.

##### Additional isolate examined.

China, Henan Province, Nanyang, in rotting wood, under a oak forest, August 2016, K.F. Liu & Z.W. Xi, NYNU 16969.

##### GenBank accession numbers.

holotype NYNU 16782^T^ (ITS: KY213803; nrLSU D1/D2: KY213826) ; additional isolate NYNU 16969 (ITS: MT133541; nrLSU D1/D2: MT133546).

##### Notes.

Two strains formed a group related to *S. ergatensis* and *Candida broadrunensis*, which represent a new species, *S. paraergatensis*. The nucleotide differences between the new species and its closest relative, *S. ergatensis*, were 1.1% substitutions in the D1/D2 domain and 0.8% substitutions in ITS region, respectively. Similarly, *S. paraergatensis* and *C. broadrunensis* displayed 0.9% substitutions in the D1/D2 domain and 2.4% substitutions in the ITS region, respectively. Physiologically, *S. paraergatensis* can be differentiated from its closest relative, *S. ergatensis* (Lachance et al. 2011), by its ability to ferment d-xylose and assimilate l-arabinose, raffinose, inulin, and d-gluconate and its inability to assimilate l-sorbose. Additionally, *S. paraergatensis* can grow in 0.1% cycloheximide and at 30 °C, but not for *S. ergatensis*.

#### Scheffersomyces anoplophorae

Taxon classificationFungiSaccharomycetalesSaccharomycetaceae

C.Y. Chai & F.L. Hui sp. nov.

BB4AB810-0320-522D-A215-757B5D6783A8

MycoBank No: 835006

Figure 4

##### Etymology.

The species name *anoplophorae* (N.L. fem. Gen. n.) refers to the genus of the host beetle, *Anoplophora leechi*.

##### Holotype.

NYNU 15730^T^.

##### Isolation data.

China, Henan Province, Nanyang, in the gut of *Anoplophora leechi*, in the People’s Park, July 2015, R.C. Ren & K.F. Liu (ex-holotype: CICC 33086; CBS 14170).

##### Description.

**Figure 4.**
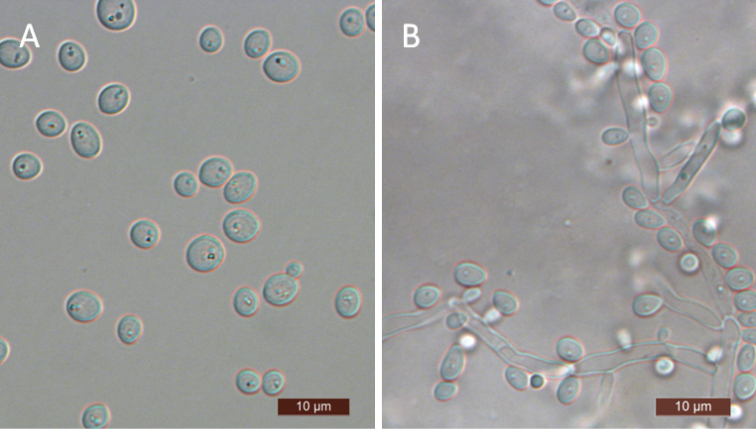
Morphology of *S. anoplophorae*. **A** budding cells **B** pseudohyphae. Scale bars: 10 μm.

The cells are spherical or ovoid (2.5–6 × 2.5–7.5 μm) and occur singly or in pairs (Fig. 4A) when placed in YM broth after 3 days at 25 °C. Budding is multilateral. After 3 days of growth on YM agar at 25 °C, the colonies are white to cream-colored, buttery, and smooth with entire margins. After 12 days at 25 °C on a Dalmau plate culture with CM agar, pseudohyphae were observed but true hyphae were not (Fig. 4B). Asci or signs of conjugation were not observed on sporulation media. Glucose, galactose, trehalose, cellobiose (weak), and d-xylose (weak) are fermented, but maltose, sucrose, melibiose, lactose, melezitose, raffinose, and inulin are not. Glucose, galactose, d-glucosamine, d-xylose, maltose, trehalose, cellobiose, salicin, glycerol, ribitol, d-glucitol, d-mannitol, d-glucono-1, 5-lactone, 2-keto-d-gluconate, 5-keto-d-gluconate, succinate, citrate, and ethanol are all assimilated. No growth was observed in l-sorbose, d-ribose, l-arabinose, d-arabinose, l-rhamnose, sucrose, methyl α-d-glucoside, arbutin, melibiose, lactose, raffinose, melezitose, inulin, erythritol, xylitol, l-arabinitol, galactitol, *myo*-inositol, d-gluconate, d-glucuronate, dl-lactate, or methanol. For the assimilation of nitrogen compounds, growth on l-lysine, glucosamine, or d-tryptophan is positive, while growth on nitrate, nitrite, ethylamine, cadaverine, creatine, creatinine, and imidazole is negative. Growth is observed at 37 °C, but not at 40 °C. Growth in the presence of 0.01% cycloheximide is positive, but growth in the presence of 0.1% cycloheximide, 10% NaCl with 5% glucose, and 1% acetic acid is negative. Starch-like compounds are not produced. Urease activity and diazonium blue B reactions are also negative.

##### Additional isolate examined.

China, Henan Province, Nanyang, in the gut of *Anoplophora leechi*, in the People’s Park, July 2015, R.C. Ren & K.F. Liu, NYNU 15733.

##### GenBank accession numbers.

holotype NYNU 15730^T^ (ITS: KU128714; nrLSU D1/D2: KU128724) ; additional isolate NYNU 15733 (ITS: MT133542; nrLSU D1/D2: MT133540).

##### Notes.

Two strains, representing *S. anoplophorae*, were clustered in a well-supported clade and were phylogenetically related to *S. stambukii* (Lopes et al. 2018). The nucleotide differences between the new species and its closest relative, *S. stambukii*, were 2.3% substitutions in the D1/D2 domain and 6.6% substitutions in the ITS region, respectively. Physiologically, the ability to assimilate d-glucosamine and the inability to assimilate d-gluconate are the primary differences between *S. anoplophorae* and its closest relative, *S. stambukii.* Additionally, *S. stambukii* can grow in 5% glucose medium with 10% NaCl, while *S. anoplophorae* cannot.

## Discussion

This study introduced and characterized three new d-xylose-fermenting species based on their morphology and phylogeny: *S. jinghongensis*, *S. paraergatensis*, and *S. anoplophorae*. While these new species share high morphological similarities with their closest relatives, *S. titanus*, *S. ergatensis*, and *S. stambukii*, they are different species due to their physiological traits and nucleotide differences in the D1/D2 domain and ITS region.

The genus *Scheffersomyces* accommodates a monophyletic group based on phylogenetic analyses of two sequence datasets (nrSSU and nrLSU) (Kurtzman and Suzuki 2010). Since it was first proposed, 18 new species and combinations have been categorized in this genus. Kurtzman and Robnett (2013) compared the species type of 70 recognized genera by analyzing sequence divergence in five-genes (nrSSU, nrLSU, *EF-1a*, *RPB1*, and *RPB2*), and determined that the genus *Scheffersomyces* is polyphyletic. *S. spartinae* was placed in a clade with *Spathaspora passalidarum*, distinct from the type species *S. stipitis*, although this placement was weakly supported by statistical analyses. However, Urbina and Blackwell (2012) suggested that the genus can be divided into three groups based on phylogenetic analysis of a multilocus dataset (nrLSU, ITS, *COXII*, and *MtSm*) with 14 described species. These results were later supported by Ren et al. (2013) and Suh et al. (2013). The results of our phylogenetic analyses of combined gene sequences (ITS and nr LSU) with all currently known species indicated that the genus is not monophyletic, but that it consists of three phylogenetically distinct groups (Fig. 1): (i) *S. stipitis* (the type species), *S. coipomoensis*, *S. shehatae*, and their related species, (ii) *S. stambukii* and *S. anoplophorae* (described in this paper) and (iii) *S. gosingicus*, *S. spartinae*, and *C. thasaenensis*. These results suggest that the genus *Scheffersomyces* should be limited to species of the group comprising the type species *S. stipitis.* The remaining two groups, which have previously been considered members of *Scheffersomyces*, could become two novel genera, although their phylogenetic relationships with other genera were not fully examined by this study (Fig. 1). As such, a careful phylogenetic analysis of *Scheffersomyces* species is required to clarify the possible heterogeneity of the genus.

Yeasts of the genus *Scheffersomyces* have been found to occupy habitats rich in xylose, including rotting wood (Ren et al. 2013; Lopes et al. 2018), wood-feeding insects (Suh et al. 2013; Urbina et al. 2013; Liu et al. 2016b), and the resulting frass (Lachance et al. 2011). We have reported the isolation and identification of several d-xylose-fermenting yeasts from wood-feeding insects and rotting wood (Ren et al. 2013; Liu et al. 2016b), including those detailed in this study. Although the samples of insects and rotting wood were collected in a relatively small geographical area in China, the d-xylose-fermenting yeasts are diverse and several of them were identified as new species (Ren et al. 2013; Liu et al. 2016b). These yeasts demonstrated several physiological traits related to the full utilization of lignocellulosic biomass, such as assimilating and fermenting xylose and/or cellobiose (Ren et al. 2013; Suh et al. 2013). Surveying d-xylose-fermenting yeasts in insects and rotting wood from various regions in different climates will help identify valuable biological and genetic resources to aid in the production of ethanol from biomass.

## Supplementary Material

XML Treatment for Scheffersomyces jinghongensis

XML Treatment for Scheffersomyces paraergatensis

XML Treatment for Scheffersomyces anoplophorae
